# Control Work

**Published:** 1897-12

**Authors:** 


### CONTROL WORK.

REFERRING to the serious epizoötic form of disease prevailing at Alvin, Galveston, and Austin, Texas, Veterinary-Surgeon Falsetter finds on the carpet-grass and rushes of the coast country a heavy deposit of black smut; nearly all fatal cases had access to these grasses. Hay- and grain-fed horses, not allowed access to these pastures were free from the disease. No traces of smut were found at Galveston, where many died on the mainland.

Live-stock Commissioners from Illinois, Indiana, Kentucky, and Tennessee met in Chicago early in November, to take action against the practice, said to be rife in Chicago, of buying and selling horses infected with glanders and cattle with Texas-tick, supposed to be the cause of splenic or Texas fever. Resolutions were unanimously adopted asking that the Secretary of Agriculture call a general convention, to meet at St. Louis or some other central point, not later than December 15th. One of the most important subjects for consideration will be the present quarantine law, which is objectionable to many of the Northern dealers.

The Experiment Station authorities at London, Ont., are endeavoring to stamp out tuberculosis in that place, and have already destroyed twenty of the twenty-eight cattle affected.

Local Boards of Health at Rochester, Utica, Saranac Lake, Connellsville, and Cortland, N. Y., demand tuberculin examinations of all cattle used for furnishing milk for sale in these towns.

